# LRP5 Regulates Development of Lung Microvessels and Alveoli through the Angiopoietin-Tie2 Pathway

**DOI:** 10.1371/journal.pone.0041596

**Published:** 2012-07-25

**Authors:** Tadanori Mammoto, Jing Chen, Elisabeth Jiang, Amanda Jiang, Lois E. Smith, Donald E. Ingber, Akiko Mammoto

**Affiliations:** 1 Vascular Biology Program, Department of Surgery, Boston Children’s Hospital and Harvard Medical School, Boston, Massachusetts, United States of America; 2 Department of Ophthalmology, Boston Children’s Hospital and Harvard Medical School, Boston, Massachusetts, United States of America; 3 The Manton Center for Orphan Disease Research, Boston Children’s Hospital and Harvard Medical School, Boston, Massachusetts, United States of America; 4 Wyss Institute for Biologically Inspired Engineering, Boston, Massachusetts, United States of America; 5 Harvard School of Engineering and Applied Sciences, Cambridge, Massachusetts, United States of America; Medical University Innsbruck, Austria

## Abstract

Angiogenesis is crucial for lung development. Although there has been considerable exploration, the mechanism by which lung vascular and alveolar formation is controlled is still not completely understood. Here we show that low-density lipoprotein receptor-related protein 5 (LRP5), a component of the Wnt ligand-receptor complex, regulates angiogenesis and alveolar formation in the lung by modulating expression of the angiopoietin (Ang) receptor, Tie2, in vascular endothelial cells (ECs). Vascular development in whole mouse lungs and in cultured ECs is controlled by LRP5 signaling, which is, in turn, governed by a balance between the activities of the antagonistic Tie2 ligands, Ang1 and Ang2. Under physiological conditions when Ang1 is dominant, LRP5 knockdown decreases Tie2 expression and thereby, inhibits vascular and alveolar development in the lung. Conversely, when Ang2 dominates under hyperoxia treatment in neonatal mice, high LRP5 and Tie2 expression suppress angiogenesis and lung development. These findings suggest that the LRP5-Tie2-Ang signaling axis plays a central role in control of both angiogenesis and alveolarization during postnatal lung development, and that deregulation of this signaling mechanism might lead to developmental abnormalities of the lung, such as are observed in bronchopulmonary dysplasia (BPD).

## Introduction

The formation of the pulmonary capillary vessels and dynamic postnatal alveolar formation are tightly coupled processes; new alveolar septa are formed with the closely associated capillaries that protrude into primitive air space to increase the surface area, which is available for gas exchange [1]–[3]. Angiogenesis, the formation of capillary network, plays a critical role in this process and a potent angiogenic growth factor, vascular endothelial growth factor (VEGF) plays an important role in postnatal lung alveolar development [4] as well as the maintenance of alveolar structures in adult lung [5], [6]. The inhibition of VEGF not only impairs pulmonary vascular network development, but also leads to the formation of immature alveoli [7]–[9]. Thus, angiogenesis is known to play crucial roles in physiological lung development [10]–[15], and deregulation of this process leads to lung pathologies such as bronchopulmonary dysplasia (BPD) [7], [9]. In addition to VEGF, the angiopoietin (Ang)-Tie2 pathway also contributes to angiogenesis [16]–[18] by modulating migration and intercellular remodeling of endothelial cells (ECs) [19]–[22]. Importantly, Tie2 and Angs are extensively expressed in lung and deregulation of this system contributes to the pathogenesis of various lung diseases including pulmonary fibrosis and asthma [23], [24]. Ang1 and Ang2 bind to their common receptor Tie2, antagonize each other and control blood vessel maturation and stabilization during angiogenesis [25], [26]; Ang1 stabilizes blood vessel formation [21], [27], whereas Ang2 destabilizes the blood vessel structure in lung injury such as BPD [11], [28], [29]. Given that the level of Ang1 dynamically increases, while Ang2 decreases in the lung after birth [30] and that tracheal aspirate samples from the BPD model mouse have high levels of Ang2 [31], [32], the Ang/Tie2 pathway may play a crucial role in postnatal lung vascular and alveolar development. However, the precise mechanisms by which the Ang/Tie2 pathway regulates lung organ morphogenesis during normal development and in pathology have not been elucidated.

Wnt signaling is also a key regulator in lung vascular development [33]–[38] and deregulation of the signaling contributes to various chronic lung diseases including BPD and pulmonary fibrosis [39]–[43]. Among Wnt signaling molecules, an essential component of Wnt ligand-receptor complex, low-density lipoprotein receptor-related protein 5 (LRP5), is known to be expressed in the lung [43] and to regulate postnatal vascular development in the retina [33], [44]–[46] and the lung [37], [38], [47]. For example, Wnt7b can bind to Fzd-1 and −10 receptors and cooperatively activate canonical Wnt signaling in the presence of LRP5 and regulate lung airway and vascular development [48]. Since both Ang-Tie2 and Wnt signaling pathways play an important role in vascular development in the lung, we have explored the link between these two pathways to elucidate the molecular mechanism underlying lung development.

In this report, we have demonstrated that LRP5 modulates vascular development in the lung by stimulating Tie2 gene expression in pulmonary microvascular endothelial cells and that the balance between Ang1 and Ang2 determines the consequences of LRP5/Tie2 signaling on angiogenesis and alveolar formation. During physiological development when Ang1 is dominant in the lung, loss of LRP5 causes impaired lung development. However, in the mouse hyperoxia-induced BPD model when Ang2 is dominant in the lung, downregulation of LRP5/Tie2 partially protected neonatal lungs from developing the pathological pictures of BPD. Although surfactant therapy, antenatal steroids, and advances in the strategies of neonatal intensive care such as ventilatory management have improved the survival of BPD patients, BPD still remains a significant complication of premature birth that is characterized by persistent respiratory problems including a prolonged need for mechanical ventilation or oxygen therapy, recurrent hospitalizations for frequent respiratory infection and distress, and long-term exercise intolerance. Many problems persist beyond childhood and cause a heavy socioeconomic toll [49]. Our findings may lead to new and better understandings of the pathogenesis of BPD, which may improve the current therapeutic strategies.

## Results

### LRP5 Controls Tie2 Expression in Lung Microvascular ECs

**Figure 1:**
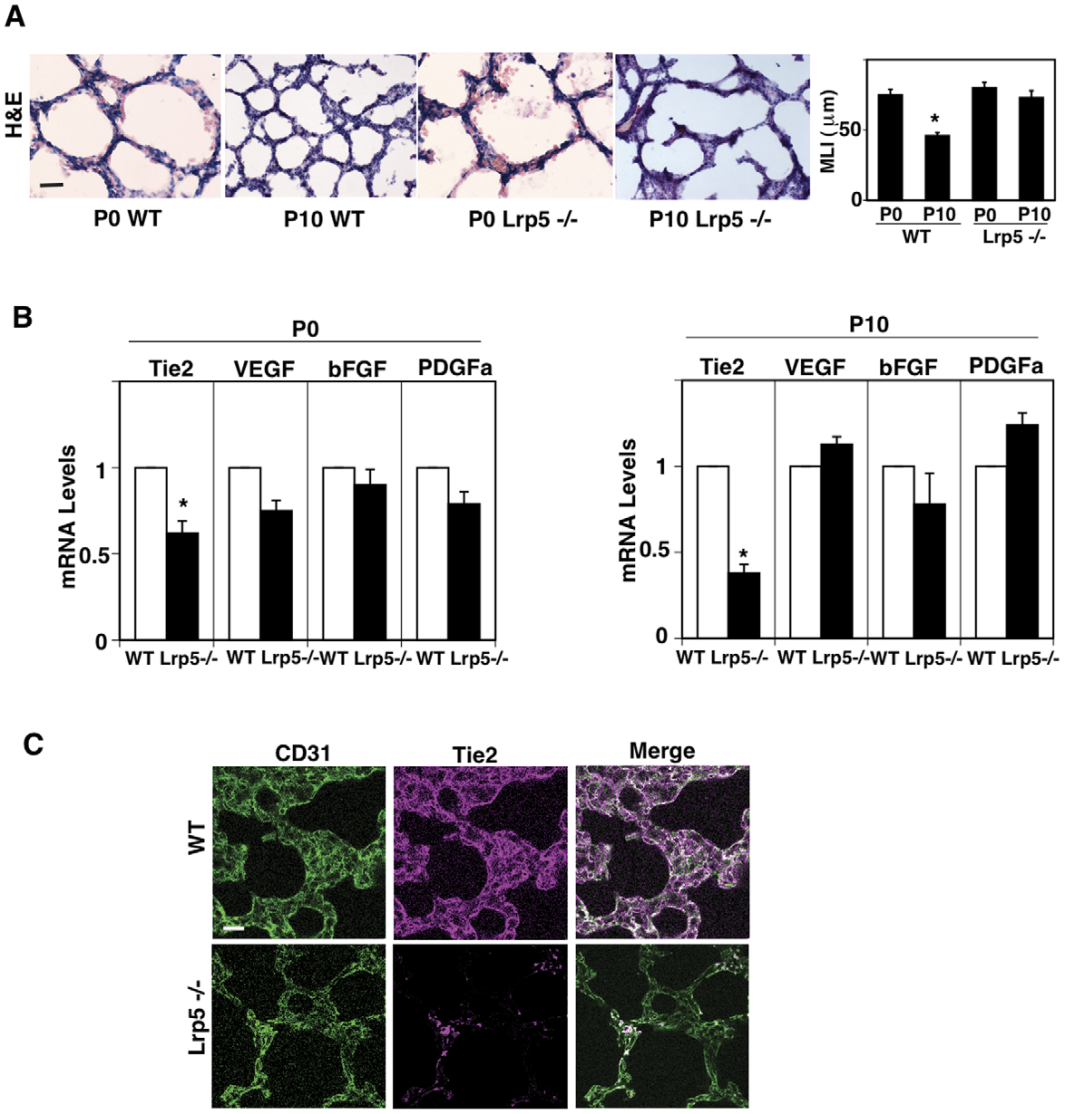
Lrp5 controls Tie2 expression and lung vascular and alveolar formation *in vivo*. **A**) H&E staining of the lungs from WT and Lrp5−/− mice (P0 and P10) (left). Scale bar, 50 µm. Graph showing the mean linear intersect (MLI) in the lungs from WT and Lrp5−/− mice (P0 and P10) (right, n = 8, * p<0.01). **B**) mRNA levels of Tie2, VEGF, bFGF and PDGFa in the lungs from WT mice and Lrp5−/− mice at P0 and P10 (n = 8, *p<0.01). **C**) Immunofluorescence (IF) micrographs showing CD31-staining vessels (green), and Tie2 expression (magenta) in the lungs of WT (upper panels) and Lrp5−/− mice (lower panels) at P10. Scale bar, 25 µm. Data are expressed as mean ± s.e.m. throughout all figures.

It has been reported that LRP5 plays important roles in lung vascular and alveolar development [37], [38], [43], [47] as well as retinal angiogenesis [33], [45], [46], [50]. Therefore, we first examined whether Lrp5 regulates postnatal lung development using Lrp5 knockout (Lrp5−/−) mice. When we examined alveolar structure in the lung using histological analysis, the subdivision of alveolar units (septation) was incomplete and alveolar spaces were large at postnatal day 0 (P0) in both wild type (WT) and Lrp5−/− mice (Fig. 1A). Although extensive septation was developed in the lungs at P10 in the WT mice (Fig. 1A), the septation was still incomplete and alveolar spaces were large in Lrp5−/− mouse lungs at P10 (Fig. 1A). When alveolar structure was characterized using the mean linear intercept (MLI) method, the MLI was significantly lower in the lungs at P10 compared to that of P0 mouse lungs in the WT mice, while there was not a significant difference between P0 and P10 in the Lrp5−/− mice (Fig. 1A). Since the Ang-Tie2 pathway contributes to angiogenesis in the lung [23], [24], [30]–[32], we examined Tie2 expression in the mouse lungs. Tie2 mRNA levels were significantly lower in Lrp5−/− mouse lungs compared to those in age-matched WT mouse lungs at P0 and P10 (Fig. 1B). However, the expression of other factors known to be involved in angiogenesis such as VEGF, bFGF and PDGFa was not significantly different between WT and Lrp5−/− mouse lungs at P0 and P10 (Fig. 1B). Immunofluorescent (IF) detection in tissue sections shows that LRP5 and Tie2 proteins are specifically expressed in ECs (CD31 positive cells) (Fig. S1, Fig. 1C) and expression of Tie2 in the ECs is significantly decreased in the lungs of Lrp5−/− mouse at P10 compared with that in the lungs of age-matched WT mouse (Fig. 1C), suggesting that LRP5 regulates septation during the postnatal period by changing the expression of Tie2 in the capillary blood vessels.

**Figure 2:**
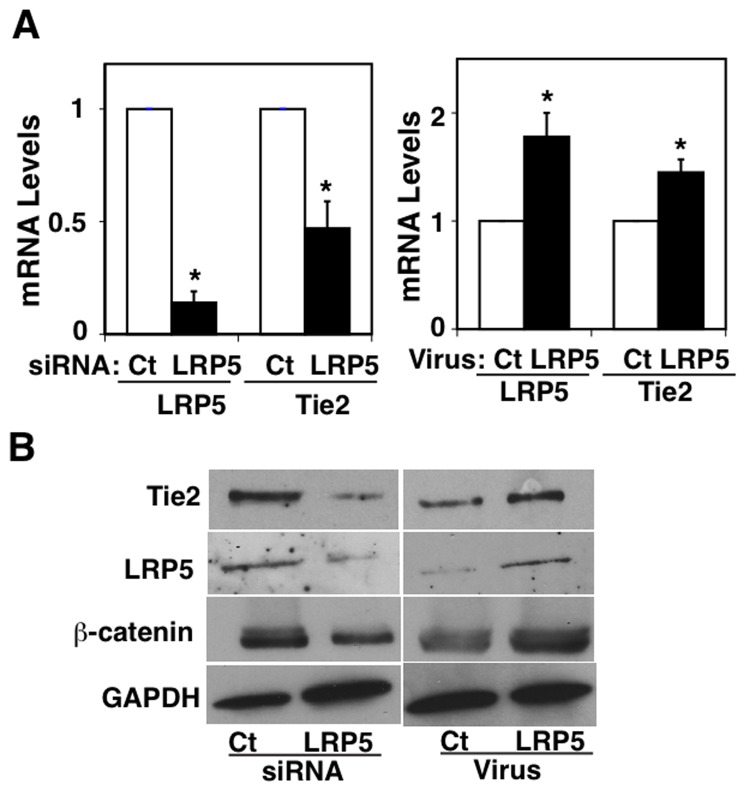
LRP5 controls Tie2 expression in ECs. **A**) Graphs showing Tie2 and LRP5 mRNA levels in L-HMVE cells treated with LRP5 siRNA (left) or retroviral vectors encoding LRP5 (right) (*p<0.01). **B**) Immunoblots showing Tie2, LRP5, β-catenin and GAPDH protein levels in L-HMVE cells treated with LRP5 siRNA (left) or retroviral vectors encoding LRP5 (right). Error bars represent s.e.m. of at least three replica experiments.

Having established that LRP5 regulates Tie2 expression in mouse lung ECs *in vivo*, we next examined whether LRP5 controls Tie2 expression in human lung microvascular endothelial (L-HMVE) cells *in vitro.* Knockdown of LRP5 using siRNA transfection decreased both mRNA and protein levels of Tie2 in L-HMVE cells by half, while LRP5 overexpression induced the opposite effect (Fig. 2A, B). Importantly, LRP5 knockdown decreased, while LRP5 overexpression increased β-catenin protein levels in L-HMVE cells (Fig. 2B), suggesting that LRP5 controls Tie2 expression in lung ECs via the canonical WNT signaling pathway, in which β-catenin is degraded when it is inactive.

### LRP5 and Tie2 Control Angiogenesis in Lung Microvascular ECs

**Figure 3:**
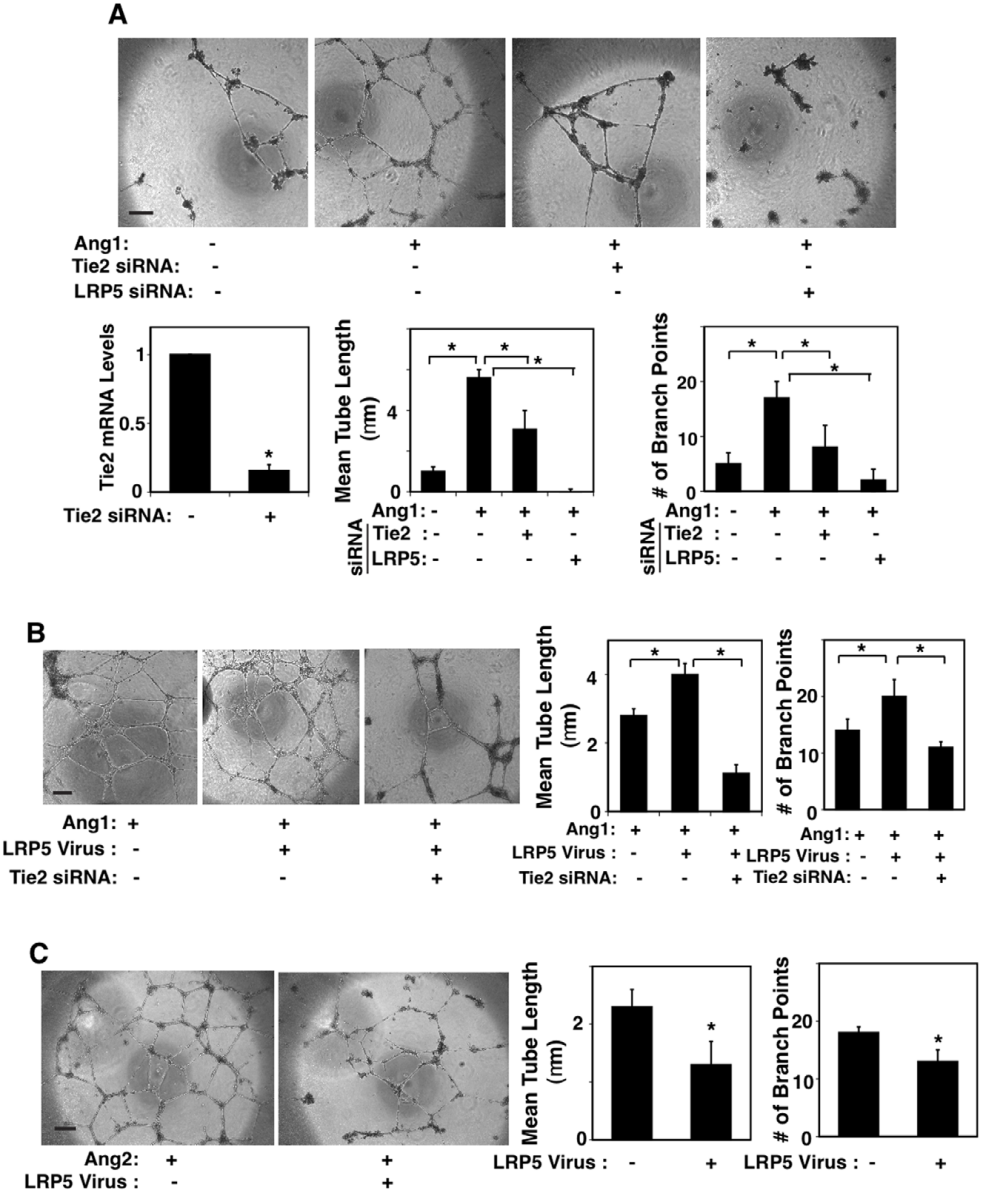
LRP5 and Tie2 control angiogenesis in L-HMVE cells. **A)** Micrographs showing *in vitro* tube formation induced by Ang1 (50 ng/ml) in L-HMVE cells (under 0.2% FBS). The cells were transfected with siRNA against Tie2 or LRP5. Scale bar, 50 µm. Graphs showing Tie2 mRNA levels in L-HMVE cells (left), the mean tube length per field (middle) and the number of branch points (right) from ten fields (*p<0.01). **B**) Micrographs showing *in vitro* tube formation induced by Ang1 (50 ng/ml) in L-HMVE cells (under 0.2% FBS) treated with retrovirus encoding LRP5 or in combination with Tie2 siRNA. Scale bar, 50 µm. Graphs showing the mean tube length per field and the number of branch points from ten fields (*p<0.01). Error bars represent s.e.m. of at least three replica experiments. **C)** Micrographs showing *in vitro* tube formation induced by Ang2 (50 ng/ml) in L-HMVE cells treated with retrovirus encoding LRP5. Scale bar, 50 µm. Graphs showing the mean tube length and the number of branch points from ten fields (*p<0.01). Error bars represent s.e.m. of at least three replica experiments.

We then studied capillary tube formation of L-HMVE cells cultured on Matrigel. Under low serum concentration (0.2%), the ECs form incomplete tube structures on the gel (Fig. 3A, left panel). The Tie2 agonist, Ang1 (50 ng/ml) enhanced the ability of the cells to form tubes (Fig. 3A, second panel). Ang1-induced tube formation was abrogated by decreased Tie2 expression levels in the ECs through knockdown of Tie2 (Fig. 3A, third panel) or LRP5 (Fig. 3A, right panel). Knockdown of Tie2 in ECs using siRNA treatment had little effect on apoptosis when examined using TUNEL assay (Fig. S2). LRP5 overexpression, which upregulates Tie2, enhanced tube formation in the presence of Ang1 and these effects were inhibited by Tie2 knockdown (Fig. 3B). Importantly, LRP5 overexpression inhibited endothelial tube formation when cultured with Ang2 (Fig. 3C). These results suggest that LRP5 regulates angiogenesis by changing the expression of Tie2 and that the balance of antagonistic Tie2 ligands, Ang1 and Ang2, determines the effects of LRP5 on angiogenesis *in vitro*.

### Lung Angiogenesis is Regulated by LRP5 and Tie2 *in vivo*

**Figure 4:**
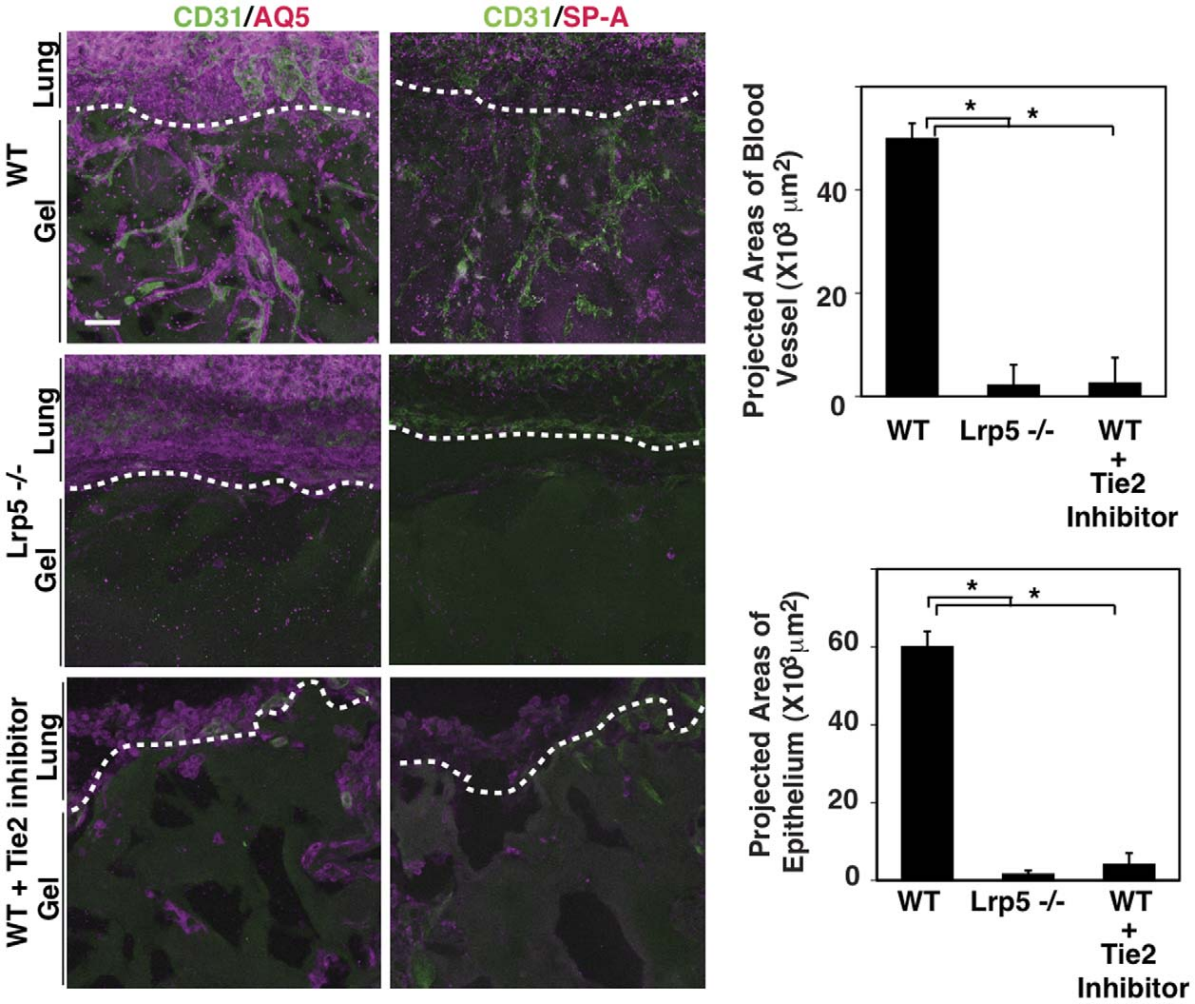
LRP5 regulates endothelial and epithelial cell recruitment in the gels implanted on mouse lungs. IF micrographs showing *in vivo* angiogenesis and alveolar formation in the Matrigel implanted on the mouse lungs. The gels were implanted on the lungs of WT mice (upper panels) or Lrp5−/− mice (middle panels). The gels with Tie2 inhibitor (bottom panels) were also implanted on the lung of WT mice. IF micrographs detecting CD31-positive blood vessels (green), AQ5- positive type I epithelium (magenta, left panels) or SP-A-positive type II epithelium (magenta, right panels). Scale bar, 50 µm. Graphs showing the projected areas of blood vessels and epithelium from ten fields (n = 8, *p<0.01). Error bars represent s.e.m.

To further evaluate the role of LRP5 on angiogenesis and alveolar formation *in vivo*, we implanted Matrigel on living young adult mouse lungs for 10 days. Vascular network formation and recruitment of both type I and II lung epithelium (as shown with their respective markers aquaporin5 (AQ5) and surfactant protein-A (SP-A)) along the vasculature were observed in the gels implanted on WT mouse lungs (Fig. 4, upper panels). However, these structures were not formed in the gels implanted on Lrp5−/− mouse lungs (Fig. 4, 2nd panels) or in the gels with Tie2 inhibitor (10 µM) (Fig. 4, 3rd panels). These findings support the hypothesis that LRP5/Tie2 signaling plays a crucial role in angiogenesis and alveolar formation.

### Hyperoxia Disrupts Lung Vascular Formation Through the LRP5-Tie2 Pathway

**Figure 5:**
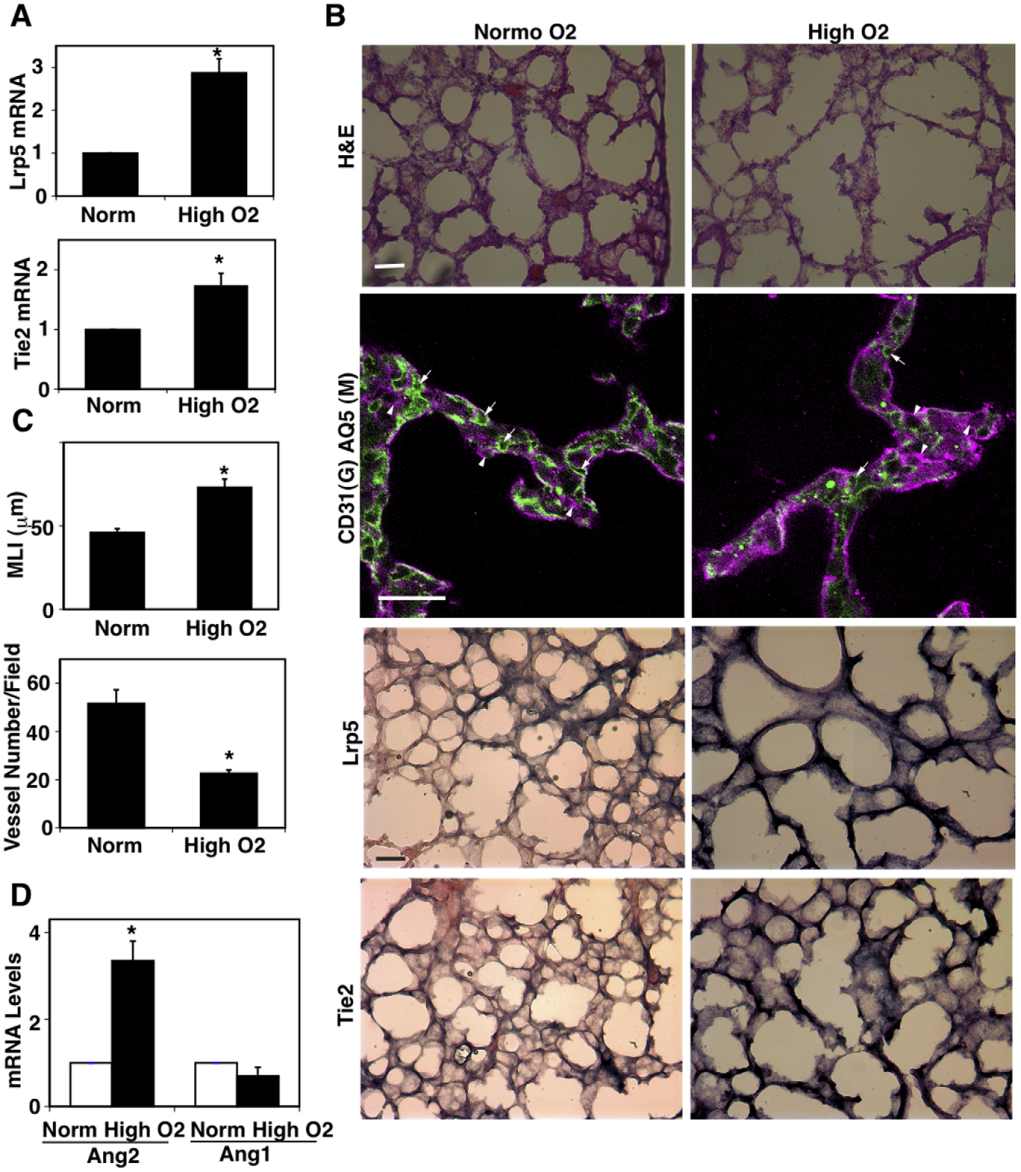
Hyperoxia regulates lung vascular formation via Lrp5 and Tie2 *in vivo*. **A**) Graphs showing mRNA levels of Lrp5 and Tie2 in neonatal mouse lungs treated with normoxia (21% O_2_) or hyperoxia (75% O_2_) for 10 days (n = 8, *p<0.01). **B**) Light (H&E-stained) micrographs (top), IF micrographs (2nd) showing formation of CD31-positive blood vessels (green, indicated by arrows) and AQ5-positive type I epithelium (magenta, indicated by arrowheads), and ISH images (3^rd^ and bottom) detecting Lrp5 and Tie2 in neonatal mouse lung treated with normoxia (21% O_2_) or hyperoxia (75% O_2_) for 10 days. Scale bars, 50 µm for top, third and bottom panels, 25 µm for second panels. **C**) Graphs showing MLI (upper) and the mean number of CD31-positive blood vessels segments per field (that is, segments between branch points) in alveolar septa (lower, n = 8, *p<0.01). **D**) Graphs showing Ang1 and Ang2 mRNA levels in neonatal mouse lungs treated with normoxia (21% O_2_) or hyperoxia (75% O_2_) for 10 days (n = 8, *p<0.01). Error bars represent s.e.m.

**Figure 6:**
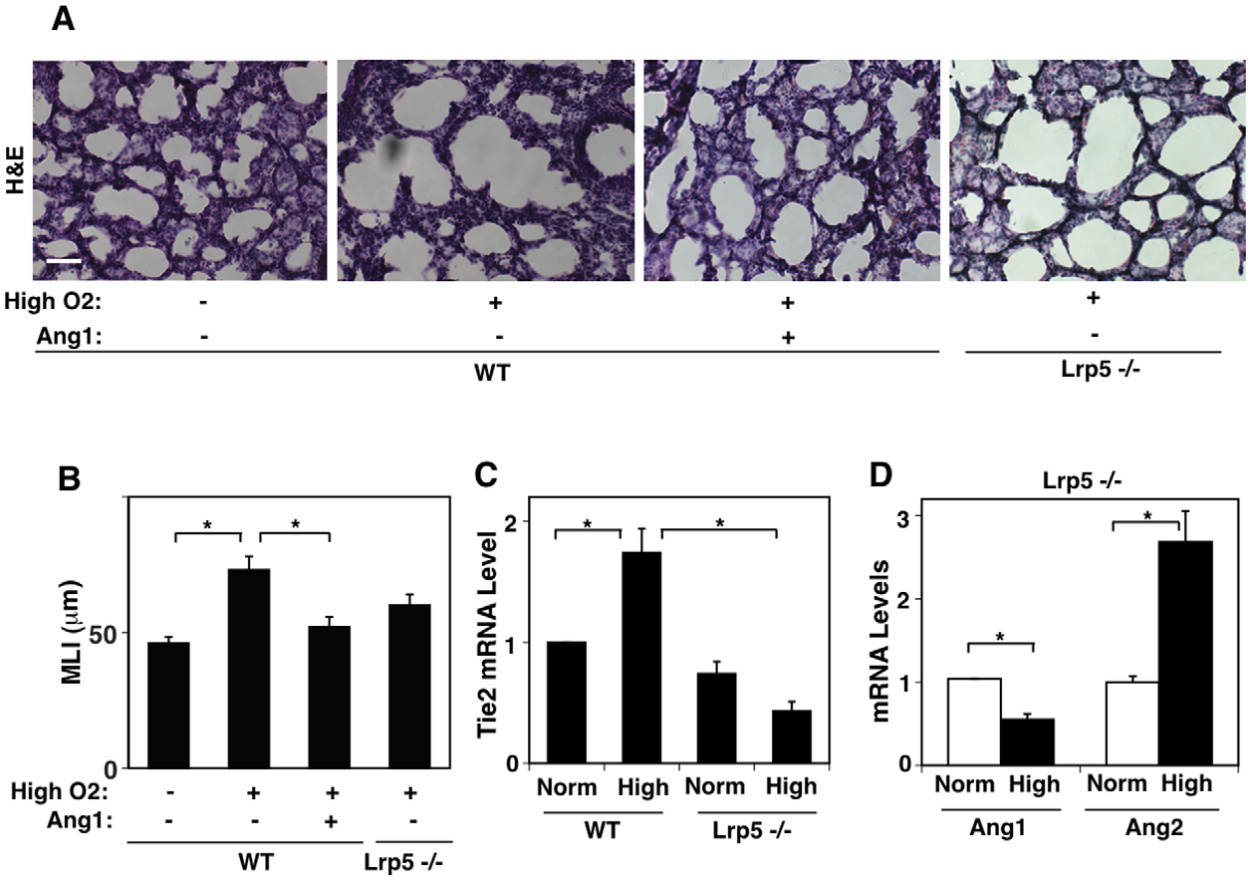
Ang1 rescues hyperoxia-induced lung injury *in vivo*. A) H&E-stained light micrographs showing the lungs of WT mice (P10) treated with normoxia (21% O_2_), hyperoxia (75% O_2_) with or without Ang1, or the lungs of Lrp5−/− mice (P10) treated with hyperoxia for 10 days. Scale bar, 25 µm. **B**) Graphs showing MLI of the lungs in each condition described in A) (n = 8, *p<0.01). **C**) Graphs showing the Tie2 mRNA levels in neonatal WT or Lrp5−/− mouse lungs treated with normoxia (21% O_2_) or hyperoxia (75% O_2_) for 10 days (n = 8, *p<0.01). **D**) Graphs showing Ang1 and Ang2 mRNA levels in neonatal Lrp5−/− mouse lungs treated with normoxia (21% O_2_) or hyperoxia (75% O_2_) for 10 days (n = 8, *p<0.01). Error bars represent s.e.m.

Our *in vitro* results suggest that the LRP5/Tie2 signaling pathway regulates lung vascular development through the balance of Ang1 and Ang2 (Fig. 3). It has been reported that exposure of neonatal mice to hyperoxia results in arrest of postnatal lung development [31], [42], [51]. When we exposed neonatal mice to high oxygen (75% O_2_) for 10 days (P1–P10), mRNA levels of Lrp5 and Tie2 in the lungs increased by three- and two-fold, respectively, compared with the age-matched normoxia (21% O_2_) treated control lungs (Fig. 5A). However, the lungs exposed to high oxygen failed to make septation, resulting in fewer and larger alveoli with less developed vascular structures that mimic the lungs of human premature infants with BPD (Fig. 5B upper and second panels, Fig. 5C). In situ hybridization (ISH) analysis confirmed that the LRP5 and Tie2 mRNA levels were higher in the lungs exposed to hyperoxia (Fig. 5B third and bottom panels). Importantly, mRNA levels of Ang2 were four-times higher in the lungs exposed to hyperoxia, while Ang1 levels remained the same compared with control lungs (Fig. 5D), consistent with the report for human premature infants with BPD [52]. These data are also consistent with our *in vitro* data showing that Ang2 disrupts capillary endothelial tube formation when LRP5/Tie2 signaling is activated (Fig. 3C). Administration of Ang1 (500 ng, i.p., every other day), an antagonist of Ang2 over the Tie2 receptor, restored impairment of the alveolar development induced by exposure to high oxygen (Figs. 6A, B) and these pathological phenotypes were also partially rescued in Lrp5−/− mice (Figs. 6A, B) in which Tie2 expression in the lung was low (Fig. 6C). Given that the exposure to hyperoxia increases the level of Ang2 in Lrp5−/− mice (Fig. 6D), hyperoxia may deregulate lung vascular and alveolar formation through the enhancement of endothelial LRP5/Tie2/Ang2 signaling in the postnatal lung.

## Discussion

**Figure 7:**
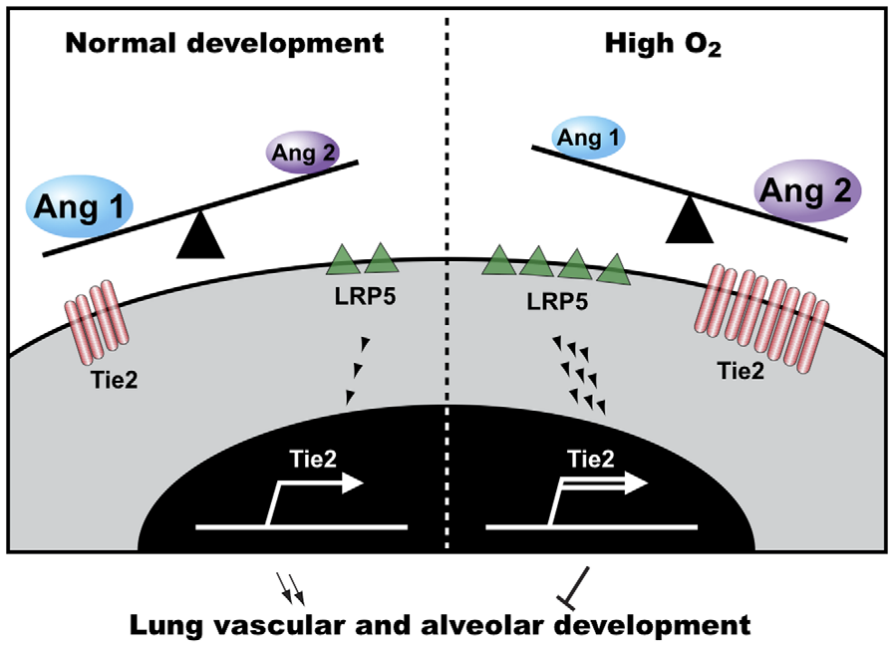
LRP5 regulates lung vascular and alveolar development through the Ang-Tie2 pathway. Angiogenesis and alveolar formation in the lung can be controlled by LRP5-Tie2 signaling depending on the Ang1/Ang2 ratio. In normal postnatal lung development where Ang1 is dominant, LRP5, an essential component of the canonical Wnt signaling pathway, increases Tie2 expression in ECs and stimulates (downward arrow symbols) neonatal lung vascular and alveolar development. However, LRP5-Tie2 signaling inhibits (T-bar symbol) vascular and alveolar development when the lungs are exposed to hyperoxia in which Ang2 is upregulated.

Here we show that angiogenesis and alveolar formation in the lung can be suppressed by both activation and suppression of Wnt signaling depending on the balance between Ang1 and Ang2. In normal postnatal lung development where the ratio of Ang1and Ang2 is in favor of Ang1, knockdown of LRP5, an essential component of the canonical Wnt signaling pathway [53], [54], decreases the expression of Tie2 in ECs and hence inhibits physiological neonatal lung vascular and alveolar formation. On the contrary, during a pathological condition such as hyperoxia-induced lung injury, in which Ang2 is dominant, activated LRP5/Tie2 signaling inhibits angiogenesis and alveolar formation in the neonatal lungs (Fig. 7). These findings suggest the opposite roles of LRP5-Tie2 signaling pathway in the regulation of lung vascular and alveolar development in physiological and pathological conditions, which depends on the balance between Ang1 and Ang2.

In addition to transport of oxygen and nutrients to the organs, angiogenic factors and ECs themselves represent a crucial source of instructive signals to non-vascular tissues during lung development [55]–[56] as well as adult lung alveolar regeneration [57], [58]. In fact, we have demonstrated that angiogenic factor receptor Tie2 and its regulator LRP5 control vascular network formation and alveolar morphogenesis in our Matrigel implantation model on the lung. Therefore, blood vessels in the lung seem to actively guide normal alveolar development through the LRP5/Tie2/Ang signaling system. However, since Tie2 knockout mice die *in utero* while Lrp5 knockout mice survive, there may be other compensatory pathways that modulate Tie2 expression in the developing lung. Furthermore, Wnt ligands such as Wnt2, Wnt5a, Wnt7b and Wnt11 are expressed in the epithelium and mesenchyme of the lung [38] [56] and activation of the Wnt pathway in these components also plays an important role in lung development [59], suggesting that lung development may be regulated by a complex series of reciprocal interactions of blood vessels and these components.

Precise spatiotemporal regulation of antagonistic Ang1 and Ang2 signaling [30] is required for postnatal lung capillary network formation [60]; Ang2 is expressed by ECs located at the leading edge of proliferating vessels and acts as a destabilizing factor in areas of vascular remodeling [61], [62], while Ang1 induces translocation of Tie2 receptors to cell-cell contact or cell-matrix interface to stabilize endothelial cell-cell junction integrity or to regulate EC migration [22], [63]. Importantly, in Tie2-deficient mice, vascular plexus fails to remodel or mature and does not develop beyond the primitive stage [64] and the endothelial cell adherence and interaction with perivascular cells and ECM are deregulated [65]. The Ang-Tie2 system in ECs also regulates ECM remodeling which is necessary for organ formation [66]. Thus, the precise regulation of Tie2 signaling through a switch of soluble ligands Ang1 and Ang2 expression plays an important role in normal postnatal lung development [21], [27] as well as in pathological conditions such as BPD [11], [28], [29]. We have found that hyperoxia increased the expression of LRP5, Tie2 and Ang2 and inhibited the lung development in neonatal mice, which was attenuated in Lrp5−/− mice in which Tie2 expression was downregulated. Thus, LRP5-Tie2 signaling has dual roles in angiogenesis and alveolar development in a context-dependent way [67], [68]. However, Ang2 treatment alone did not disrupt lung vascular and alveolar formation as we observed in hyperoxia treated mice (data not shown). Other factors together with Ang2 may contribute to the pathogenesis of hyperoxia-induced lung injury. Given that LRP5 is required for embryonic and postnatal development such as bone and eye through vascular network formation [33], [44]–[46], [69], the LRP5/Tie2/Angs signaling systems may also contribute to the development of these organs.

In summary, here we first demonstrate that the LRP5-Tie2 pathway controls neonatal vascular and alveolar development in an Angs-dependent way. This pathway also contributes to the pathogenesis of hyperoxia-induced lung injury in the neonate. Thus, modulation of the LRP5-Ang/Tie2 system is likely to lead to the identification of new therapeutic targets for neonatal pulmonary diseases such as BPD.

## Materials and Methods

### Materials

Anti-Tie2 monoclonal, anti-LRP5, -β-catenin, -AQ5, β-GAL polyclonal antibodies were from Abcam. Anti -CD31 monoclonal antibodies were from BD Biosciences (San Diego, CA). Anti–GAPDH monoclonal antibody was from Chemicon. Anti-SP-A polyclonal antibody was from Millipore. Ang−1 and −2 were from R & D systems (Minneapolis, MN). Tie2 inhibitor was from BioMol/Enzo Life Science. L-HMVE cells (Cambrex, Walkersville, MD) were cultured in EBM2 medium containing 5% FBS and growth factors (VEGF, bFGF and PDGF) for all experiments except the *in vitro* tube form assays with Ang1 and Ang2 in which we used EBM2 with 0.2% serum.

### Animals Experiments

All animal studies were reviewed and approved by the Animal Care and Use Committee of Boston Children’s Hospital. Unless otherwise indicated, C57BL/6 mice (stock no. 664; Jackson Laboratory) were used for the study. The Lrp5−/− mice (stock no. 005823; Jackson Laboratory) were developed by Deltagen Inc [70]. For hyperoxia-induced lung injury, newborn pups were subdivided into two groups and exposed to 75% O_2_ in a Plexiglas chamber or 21% O_2_ within 24 hours of birth [51]. Dams were rotated from hyperoxia to room air every 24–48 hours to prevent excessive oxygen toxicity to the adult animals. At P10, lungs were harvested for histological and mRNA analysis.

### Lung Morphometry

Lungs were fixed with 4% paraformaldehyde solution through the trachea under a constant pressure of 20 cm H_2_O. The trachea was then ligated, and the lungs were immersed in fixiative overnight at 4C. Lungs were processed and embedded in OCT compound [70]. Serial step sections, 5 µm in thickness were taken along the longitudinal axis of the lobe. The fixed distance between the sections was calculated so as to allow systematic sampling of 10 sections across the whole lobe. Lungs were analyzed by hematoxylin and eosin (H&E) staining, IHC and ISH [71], [72]. The Lrp5 gene was targeted mutated and a bacterial lacZ gene was inserted into the gene such that the endogenous Lrp5 gene promoter drives expression of beta-galactosidase. To visualize lacZ gene, immunostaining was performed with anti-β-GAL antibody on lung tissue sections. Probes for ISH were produced using primers: Lrp5 sense (5′-GCGGAGTGAAGCTGGAGTCC-3′) and antisense (5′-GTAATACGACTCACTATAGGGCCTCGCATGGTGTGTGGAAGGG-3′), Tie2 sense (5′-AGGAGTCTGGGTCTGCAG-3′) and antisense (5′-GGGATCCGGATTGTTTTTGG-3′) as described previously [72]. Alveolar structures were quantified on a motorized microscope stage using mean linear intercept (MLI) method [73].

### Plasmid Construction, Gene Knockdown, Biochemical and Molecular Biological Methods

For virus construction, the full-length myc-Lrp5 (mouse) was constructed by PCR using full length plasmids from Open Biosystems as a template and subcloned into the pOC retroviral backbone vector at the NotI/BamHI sites. The generation of viral vectors was previously described [71], [74]. Gene knockdown was performed using the RNA interference technique [71]. siRNA for human LRP5 (5′-GCCCUACAUCAUUCGAGGAAU-3′ and 5′-AUUCCUCGAAUGAUGUAGGGC-3′) was purchased from Sigma Genosys (St. Louis, MO). siRNA for human Tie2 was a smart pool siRNA from Dharmacon. As a control, siRNA duplex with irrelevant sequence (QIAGEN) was used. Quantitative reverse transcription (qRT)-PCR was performed with the Quantitect SYBR Green RT-PCR kit (QIAGEN) using ABI 7300 real time PCR system (Applied Biosystems, Foster City, CA). β2 microglobulin or cyclophilin controlled for overall cDNA content. The primers used are shown in Table S1.

### Cell Analysis Methods

For the in vitro angiogenesis assay, L-HMVE cells (10^4^ cells per 150 µl of EBM-2 with 0.2% serum) were plated on Matrigel (BD Biosciences), and incubated for 12–16 h in the presence of Ang1 or Ang2 (50 ng/ml); tube formation was assessed using total tubule length as well as the number of tubule branch points in ten random fields [71]. The cell apoptosis was assessed using TUNEL assay (Roche).

### *In vivo* Matrigel Implantation Assay

The cross-linked Matrigel with specific elasticity was cast in PDMS molds as described previously [71] and the molds were implanted on the lungs of living mice. The mice were anesthetized with tribromoethanol (Avertin) and intubated with a plastic 21-gauge cannula for mechanical ventilation (Harvard Apparatus). An incision was performed in the fifth, left intercostal space and extended to the pleural cavity. After peeling the areas of visceral pleura with the fine forceps (0.5×0.5 mm square area), the plugs were glued over the area using PeriAcryl®90 (Glustitch). After confirming homeostasis, the thorax and skin were closed with silk suture and the mechanical ventilation was terminated to recover the mice. After 10 days, the mice were euthanized and the molds containing the gel were harvested for histological analysis. Immunostaining was performed on the cryosectioned samples as described [71]. Stacks of optical sections (20 µm thick) were compiled to form three-dimensional images using Volocity 4.4 (Improvision, PerkinElmer).

### Statistical Analysis

Error bars (SEM) and *p* values were determined from the results of three or more repeated experiments. The unpaired T test after ANOVA was used for analysis of statistical significance.

## Supporting Information

Figure S1 **LRP5 localizes in endothelial cells in the neonatal lung.** IF micrographs showing CD31-positive blood vessels (green) and LRP5 expression (magenta) in the lungs of WT mice at P10. Scale bar, 20 µm.
(TIF)

Figure S2 **Tie2 siRNA has no effects on apoptosis in L-HMVE cells.** IF micrographs showing TUNEL staining of control and Tie2 siRNA-treated L-HMVE cells. Scale bar, 50 µm.
(TIF)

Table S1 **The sequences for primers for qRT-PCR.**
(DOC)
