# Supplementary figures and images for: LRP5 Regulates Development of Lung Microvessels and Alveoli through the Angiopoietin-Tie2 Pathway
Source: PLoS One. 2012 Jul 25;7(7):e41596. doi: 10.1371/journal.pone.0041596 (PMC3404972; doi:10.1371/journal.pone.0041596)

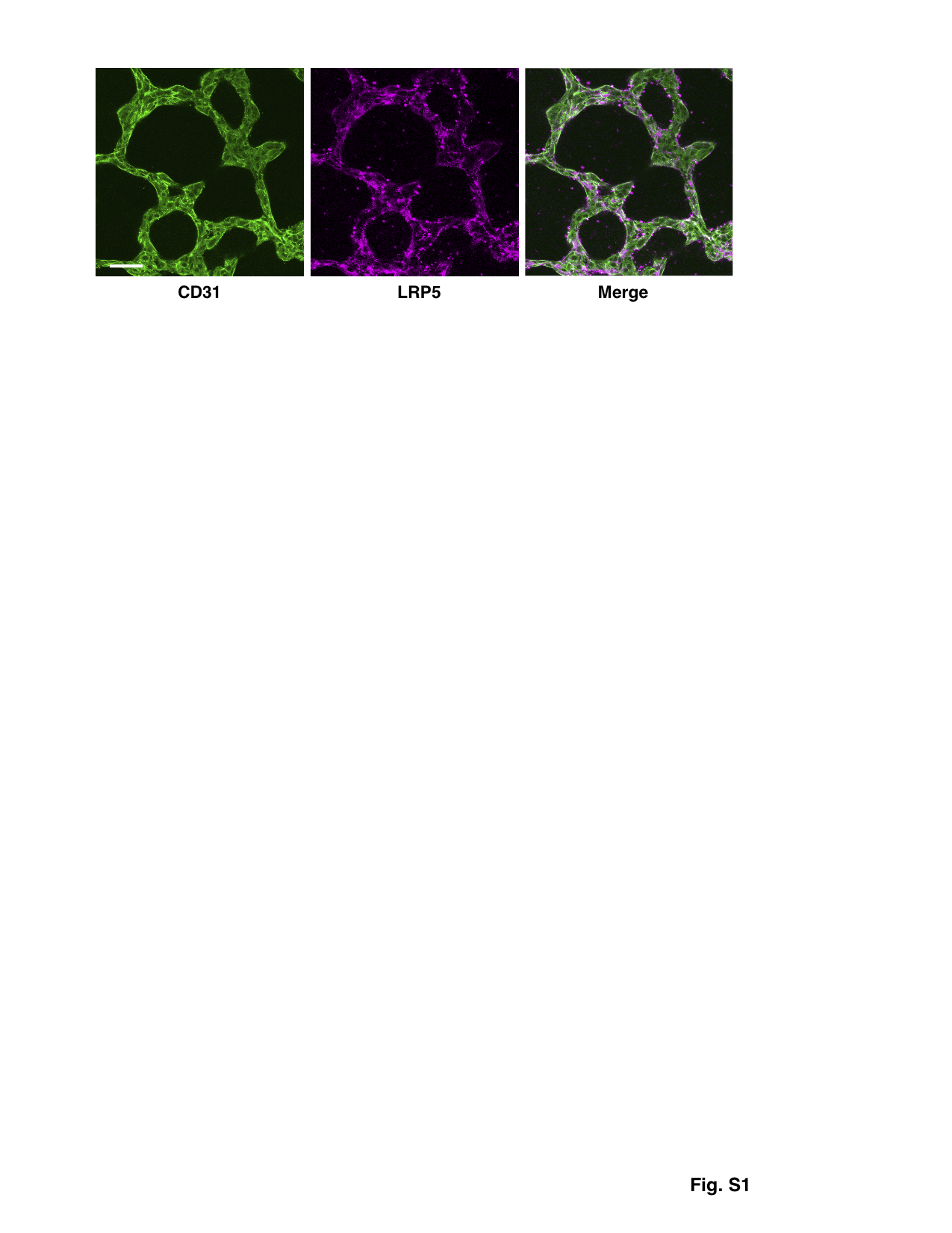

Supplement: Figure S1 — LRP5 localizes in endothelial cells in the neonatal lung. IF micrographs showing CD31-positive blood vessels (green) and LRP5 expression (magenta) in the lungs of WT mice at P10. Scale bar, 20 µm. (TIF) [file pone.0041596.s001.tif]

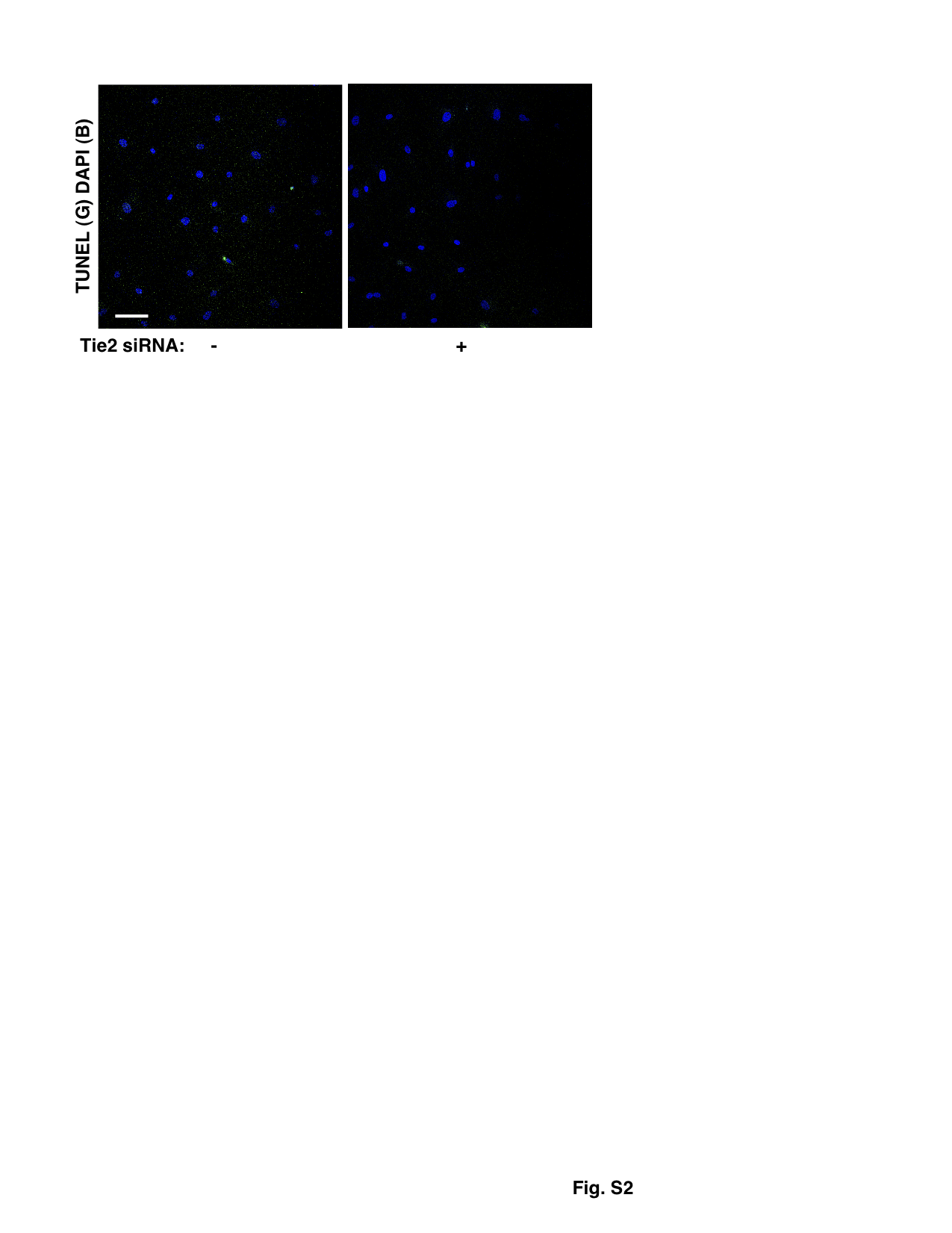

Supplement: Figure S2 — Tie2 siRNA has no effects on apoptosis in L-HMVE cells. IF micrographs showing TUNEL staining of control and Tie2 siRNA-treated L-HMVE cells. Scale bar, 50 µm. (TIF) [file pone.0041596.s002.tif]
