# Supplementary material for: LRP5 Regulates Development of Lung Microvessels and Alveoli through the Angiopoietin-Tie2 Pathway
Source: PLoS One. 2012 Jul 25;7(7):e41596. doi: 10.1371/journal.pone.0041596 (PMC3404972; doi:10.1371/journal.pone.0041596)
Supplement: Table S1 — The sequences for primers for qRT-PCR. (DOC) [file pone.0041596.s003.doc]

**Table S1. The sequences for primers for qRT-PCR**

|  | Forward | Reverse |
| --- | --- | --- |
| Human Tie2 | 5’-TGCCACCCTGGTTTTTACGG-3’ | 5’-TTGGAAGCGATCACACATCTC-3’ |
| Human LRP5 | 5’-GACCCAGCCCTTTGTTTTGAC-3’ | 5’-TGTGGACGTTGATGGTATTGGT-3’ |
| Human β2 microglobulin | 5’-GAATGGAGAGAGAATTGAAAAAGTGGAGCA-3’ | 5’-CAATCCAAATGCGGCATCTTCAAAC-3’ |
| Mouse Tie2 | 5’-CAGCTTGCTCCTTTATGGAGTAG-3’ | 5’-AGACACAAGAGGTAGGGAATTGA-3’ |
| Mouse Lrp5 | 5’-AAGGGTGCTGTGTACTGGAC-3’ | 5’-AGAAGAGAACCTTACGGGACG-3’ |
| Mouse Ang1 | 5’-GGATGTGCTGTCTAGGCAGAA-3’ | 5’-TTCATGTTCCGGCTTTCCTTT-3’ |
| Mouse Ang2 | 5’-TTAGCACAAAGGATTCGGACAAT-3’ | 5’-TTTTGTGGGTAGTACTGTCCATTCA-3’ |
| Mouse Vegf | 5’-GCACTGGACCCTGGCTTTACTGCTGTA-3’ | 5’-GAACTTGATCACTTCATGGGACTTCTGCTC-3’ |
| Mouse Pdgfa | 5’-TGTGCCCATTCGCAGGAAG-3’ | 5’-GAGGTATCTCGTAAATGACCGTC-3’ |
| Mouse bFgf | 5’-TGGTGACCACAAGCTGAATG-3’ | 5’-TCCCTTGATAGACACAACTCCTC-3’ |
| Mouse cyclophilin | 5’-CAGACGCCACTGTCGCTTT-3’ | 5’-TGTCTTTGGAACTTTGTCTGCAA-3’ |

Table S1
